# Neurophysiological Correlates in Patients with Syringomyelia and Chiari Malformation: The Cortico-Diaphragmatic Involvement

**DOI:** 10.3390/jcm11175080

**Published:** 2022-08-29

**Authors:** Dario Cocito, Erdita Peci, Diego Garbossa, Palma Ciaramitaro

**Affiliations:** 1 Department of Neuroscience “Rita Levi Montalcini”, University of Torino, 10126 Torino, Italy; 2 ICS Maugeri, 10124 Torino, Italy; 3 CRESSC-Interregional Centre of Expertise for Syringomyelia and Chiari, Città della Salute e della Scienza University Hospital, 10126 Torino, Italy

**Keywords:** Chiari malformation, syringomyelia, neurophysiology, central motor conduction time, phrenic nerve, diagnosis

## Abstract

*Purpose.* Brainstem syndromes have frequently been reported in Chiari syndrome and in syringobulbia; previous studies have shown that determining the central motor conduction time (CMCT) along the circuit of the phrenic nerve makes the assessment of the voluntary control of the respiratory pathway possible. In our study, we evaluated the transcranial magnetic stimulation (TMS) of the phrenic nerve in patients affected by Chiari syndrome and/or syringomyelia (Syr) with the aim of identifying subclinical neurophysiological alterations. *Methods.* One hundred patients (75 females; average age: 51 ± 13.08 SD; range: 18–76) affected by Chiari syndrome and/or Syr without dyspnea were selected. The magnetic stimulation of the second motor neuron correlating with the phrenic nerve was performed using cervical magnetic stimulation (C5-MEP); the cortical MEP after magnetic stimulation (Cz-MEP) was recorded by magnetic stimulation of the motor cortex (areas corresponding to the diaphragm). The CMCT was calculated. The differences between the patients and controls were calculated (Student’s *t* test). *Results.* In 13% of the patients, the Cz-MEP were absent bilaterally, and the CMCT was not evaluable. In all these cases, bulbar/cervical Syr was present at MRI; in 10 of them, this was associated with CM1. A bilateral response was obtained in all the other patients (87%), and the CMCTs were normal. All the patients with alterations/absence of Cz-MEP presented bulbar/cervical Syr at MRI. The C5-MEP latency was prolonged or absent in 48%; of these, 84% presented bulbar/cervical Syr associated with CM1 at MRI. The C5-MEP latency values were significantly higher in the group of patients. *Conclusions.* Neurophysiological alterations of the diaphragmatic pathway were recorded in a group of Chiari syndrome and, particularly, in bulbar/cervical Syr. Future studies with larger cohorts of patients are needed to further assess the specific role of the TMS of the phrenic nerve in CM/Syr patients.

## 1. Introduction

Chiari 1 malformation (CM1) is a neurological condition defined as a displacement of the cerebellar tonsils 5 mm or more below the foramen magnum (McRae line) into the cervical canal [1]. The malformation may involve the brainstem, with cervico-medullary kinking, and is frequently associated with syringomyelia-syringobulbia (Syr), characterized by fluid-filled cavities within the spinal cord and/or the bulb. Due to the breaking of neural connections in the spinal cord, Syr can lead to spinal paralysis, anesthesia, central neuropathic pain, dysautonomia and bulbar signs. About 50% of Syr patients have severe neurological damage and chronic-progressive disability with a complete loss of independence. Prognostically speaking, even more unfavorable is the presence of syringobulbia (involving the swallowing and breathing bulbar centers), a rare entity, often associated with a cervical or holochord Syr. The brainstem’s involvement in syringobulbia is characterized by an abrupt onset of symptoms; dyspnea and respiratory arrest (with an incidence of 9.4%) were recently reported in a systematic review of 53 cases [2].

Since the advent of MRI, the estimated prevalence of Syr has ranged from 1.9 to 5.9 per 100.000; that of symptomatic Syr has been 3.9 per 100.000; and the incidence of Syr has ranged from 1.06 to 10 per 100.000. A diagnosis of Syr (“clinically defined”) is made in the presence of syrinx/syringobulbia at MRI in addition to spinal/bulbar signs related to the syrinx level [3]. The estimated prevalence of CM1 in adults is 8.7 per 100.00; that of Chiari syndrome is 3.5 per 100.00. The symptomatic CM1 is defined as Chiari syndrome (clinical definition). Symptomatic Chiari syndrome is more common in adults than in children, particularly in CM1 with syringomyelia (60%); conversely, only 25% of isolated CM1 are symptomatic. The clinical diagnostic criteria include “headache caused by CM1” and neurological symptoms and signs related to the brainstem (i.e., nystagmus, dysphagia and sleep apnea), cerebellar (ataxia) and spinal cord dysfunction (i.e., muscles hypotrophy, sensory and motor deficits and autonomic disorders), as well as otoneurological deficits (e.g., dizziness, disequilibrium, sensations of alteration in ear pressure, hypoacusia or hyperacusia, nystagmus and oscillopsia). The presence of at least two of the clinical criteria is enough to judge symptomatic Chiari [3]. Brainstem syndromes have frequently been reported in Chiari syndrome and in syringobulbia; central sleep apnea syndromes (CSASs) were evidenced in 5–8% of a cohort of 600 CM1 adults by polysomnography [4], and the same frequency was reported in a large pediatric study [5]. It is therefore possible that CM1 and/or Syr patients present clinical or subclinical respiratory dysfunctions.

Multiple studies underline the importance of motor-cortical impulses directed at the respiratory muscles, which exert elaborate control over voluntary breathing in humans: the supplementary motor area (SMA) and the primary motor cortex (M1) are the sources of these cortical inputs. Neuroimaging studies often report the co-activation of the SMA with the M1 during various respiratory functions and in particular during forced inspiratory acts in humans.

Given the obvious voluntary control of respiration in speaking and singing, it is possible that the voluntary cortico-spinal circuit can modulate the excitability of the spinal motoneurons of the phrenic nerve [6]. A loss of or reduced axonal functionality of this circuit could weaken the patient’s response in cases of respiratory distress. Previous studies have shown that analyzing the central conduction time along the circuit of the phrenic nerve is a particularly suitable technique for assessing the voluntary control of the respiratory pathway.

Transcranial magnetic stimulation (TMS) in humans has been proven to be effective in the study of diaphragmatic motor-evoked potentials (MEPs); it is an easy, non-invasive and painless method for studying respiratory supraspinal pathway excitability in humans and animals [7] The cortico-diaphragmatic pathway was investigated by means of TMS in definite amyotrophic lateral sclerosis (ALS) without clinical signs of respiratory impairment. This test was a sensitive measure for revealing subclinical diaphragmatic impairment [8].

In our study, we evaluated, through TMS of the phrenic nerve circuitry, 100 patients affected by Chiari syndrome and/or syringomyelia and 30 healthy controls with the aim of identifying subclinical neurophysiological alterations affecting the circuitry.

## 2. Methods

### 2.1. Patients and Methods

#### 2.1.1. Normal Controls

The study included 30 healthy subjects, 11 males and 19 females, with a mean age of 48 ± 13.7 years (range: 23–75 years).

#### 2.1.2. Patients

One hundred patients (75 females; average age: 51 ± 13.08 SD; range: 18–76) affected by Chiari syndrome and/or syringomyelia without dyspnea were selected for the study. The diagnosis was based on the patient’s history, clinical examination and magnetic resonance imaging, according to the standardized diagnostic criteria [3,4]. All the patients presented typical symptoms, i.e., “cough”, headache and neck pain, associated with signs of brainstem compression, cerebellar syndrome and myelopathy. Clinical myelopathy can result from direct compression of the upper cord, by herniated cerebellar tonsils, or can be due to the presence of an intraspinal syrinx; similarly, siringobulbia can present brainstem signs. In Table 1, the demographic, clinical and neuroradiological data of our cohort are summarized.

All the patients presented normal values of routine respiratory function (forced vital capacity, and forced expiratory volume at the first, second and peak expiratory flows). The study protocol was approved by the Institutional Ethics Committee (protocol n.7837, 1 February 2010 and protocol n.52554, 20 May 2015) and informed consent was provided by the patients.

### 2.2. Neurophysiological Tests

Magnetic stimulation was performed using a Cadwell MES 10 stimulator, producing a maximal magnetic field of 2.3 T. A standard stimulation procedure was performed using a round coil that was 9 cm in diameter, and the recordings were conducted from the hemidiaphragm [8,9,10]. The surface recording electrodes were applied 5 cm on top of the tip of the xyphoid process (G1) and on the costal margin 16 cm from the G1 electrode ipsilaterally; the ground electrode was positioned on the ipsilateral upper arm. The stimulus intensity was adjusted to obtain the largest reproducible responses (range 65–100% stimulator output).

Stimulation of the second motor neuron correlating with the phrenic nerve was performed using cervical magnetic stimulation [11]: the round coil was kept flat against the upper cervical vertebral column with the handle pointing towards the feet, and the head of the patients was slightly bent forward. The largest amplitude of the spinal motor-evoked potential (C5-MEP) was generally obtained 1–2 cm above the C5 spinal vertebra. For magnetic stimulation of the motor cortex, the coil was applied to the scalp in the region overlying the vertex for the stimulation of the areas corresponding to the diaphragm (Cz-International 10–20 EEG system) to obtain a cortical motor-evoked potential after magnetic stimulation (Cz-MEP). The magnetic pulse was biphasic; thus, the coil’s orientation had no significance. The stimulus was applied during a quiet inspiratory effort, in order to obtain the largest amplitude of the evoked potential out of the five to ten responses recorded. The latency of the Cz-MEP was measured at the onset of the first negative deflection of the evoked response. The amplitude of the Cz-MEP was not considered due to the large variability even under normal conditions [8,9]. The central motor conduction time (CMCT) was calculated by subtracting the latencies of the spinal-evoked potentials from those obtained after cortical stimulation [CMCT = (Cz-MEP latency − C5-MEP latency)].

The findings from the magnetic stimulation study were considered abnormal if any of the parameters exceeded the upper limits of our normal values (mean + 2 SD), or if there was no response. The results obtained were thus compared with the normal values collected in our laboratory in a group of age-matched controls. Abnormal values from the diaphragm (upper limits) were: Cz-MEP latency < 15.3 ms; C5-MEP latency < 9.3 ms); CMCT < 7.6 ms.

### 2.3. Statistical Analysis

The results are expressed as the mean ± SDs. If a response was absent, a value of zero was assigned. The differences between the CM1/Syr patients and the controls were calculated by means of unpaired Student’s t tests. Values of less than 0.05 were considered significant.

## 3. Results

### 3.1. Normal Controls

In all the controls, we were able to record the Cz-MEP and C5-MEP bilaterally from the diaphragm muscle. In Table 2, we report the main electrophysiological parameters recorded.

### 3.2. Patients

#### 3.2.1. Cortical Magnetic Stimulation

In 13 patients (13%), the Cz-MEPs were absent bilaterally, despite C5-MEPs being present in 6 of them, but with pathological latency. Therefore, it was not possible to calculate the CMCTs in this group of patients. In all these cases, bulbar/cervical Syr was present at MRI; in 10 of them, it was associated with CM1. Bilateral responses were obtained in all the other patients (87%), and the CMCTs were normal. The Cz-MEP latency was prolonged or absent for 20 patients (20%).

All the patients with alterations/absence of Cz-MEP presented bulbar/cervical Syr at MRIs. Moreover, the Cz-MEP latency values were not significantly prolonged in the patient group (*vs*. *normal controls, p* > 0.05). In Table 2, the findings obtained by the surface recording from the diaphragm after transcranial and spinal magnetic stimulation are reported. Significant values (*p* < 0.05 * and *p* < 0.005 **) are highlighted in bold. 

#### 3.2.2. Cervical Magnetic Stimulation

The C5-MEP was bilaterally present in 93 patients. The C5-MEP latency was (mono or bilaterally) prolonged or absent in 48 patients (48%); of these, 40 (84%) presented bulbar/cervical Syr associated with CM1 at MRI. Moreover, the C5-MEP latency values were significantly prolonged in the group of patients (Table 2).

## 4. Discussion

The present study investigated the cortico-diaphragmatic pathway by means of magnetic stimulation in a sample of 100 CM1/Syr patients without signs and symptoms of respiratory failure. Gandevia and Rothwell, in 1987 [6] first described the activation of the human diaphragm from the motor cortex with percutaneous electrical stimulation; the TMS proved to be a similarly reliable technique with the advantage of causing less pain to the patient [12,13]. The lives of mammals depend on the autonomic genesis of the respiratory rhythm on parts of the brainstem. Suprapontine commands modulate this function according to various circumstances. These include the influence of the limbic system on respiration, as shown by the respiratory effects of different emotions and by projections of the phrenic nerve at the cingulate gyrus [11].

Using the TMS, Sharshar et al. [14] provided functional evidence of the existence of spinal projections of the SMA to phrenic motor neurons in humans. The SMA projects fibers to both the M1 and the intermediate and ventral areas of the spinal cord in macaques. Subsequently, the existence of functional connections between the SMA and the diaphragm area on the M1 was proven, in addition to the already known connections between the SMA and spinal circuits of the diaphragm.

All these studies have highlighted the fact that, in addition to a bulbar automatic control of respiration act, there is a complex series of “voluntary” upper cortical interferences. It is possible that CM1 associated or not with Syr can cause alterations both of the cortico-diaphragmatic circuit correlated with the phrenic nerve and at the level of the spinal pyramidal pathways, as well as at the level of the spinal motor neurons.

Among the causative mechanisms involved in the pathophysiology of the abnormalities in respiratory control are both a compression of the phrenic motor neurons in the anterior horn of the medulla by the associated syringomyelia and the direct bulbar compression of the respiratory center by a downward displacement of the cerebellar tonsils [15]. Acute respiratory failures are very uncommon and a few cases as first manifestations of CM are reported in the literature [16,17,18]. Furthermore, delayed diagnosis can potentially lead to respiratory failures (central and obstructive sleep apnea, prolonged expiratory apnea with cyanosis, aspiration pneumonia) and death [2,15,16,17,18,19].

In our study, we showed that a percentage of patients, even in the absence of clinical/instrumental signs of respiratory failure, presented alterations of the motor conduction correlated with the phrenic nerve. In 48% of the cases, these alterations affected the C5-MEP, while in 20% of the cases they resulted in the absence or delay of Cz-MEP. 

By contrast, the CMCTs in the C5-cortex tracts were normal in all the patients for which Cz-MEPs were recorded. Alterations of the Cz-MEP and C5-MEP were clearly prevalent in patients with cervical syringomyelia/syringobulbia, most associated with CM1.

The cortical-spinal tracks directed to the C3-C4-C5 metamers include the fibers to the phrenic nerve, which provide the motor function of the diaphragm; these fibers are responsible for the voluntary control of breathing. Therefore, the electrophysiological abnormalities that we recorded in patients affected by cervical/bulbar Syr could impact the diaphragm function. The normality of the CMCTs in most of our patients associated with a high percentage of alterations of the C5-MEP might indicate the prevalence of axonal dysfunction in the II motor neuron correlated with the phrenic nerve, rather than damage to the pyramidal pathways. Such a hypothesis is supported by the presence of a CM1 associated with a bulbar/cervical Syr in the vast majority of these patients.

To our knowledge, this is the first study performed on Chiari and Syr patients to investigate the cortico-diaphragmatic pathway by TMS. This neurophysiological method is not relevant for the early diagnosis in CS and Syr, but it may suggest the compromission of the diaphragm function (which in our patients was still subclinical) in the presence of syringomyelia-syringobulbia. Even though further studies are much needed, these data could be an important element in defining the clinical staging of the patient.

A potential source of bias in our study is the absence of an instrumental gold standard with which to validate the electrophysiological surveys; collaborative multicenter research studies, comparing neurophysiological data with advanced neuroradiologic techniques, e.g., diffusion tensor imaging (DTI-MRI), could be proposed to study the clinical significance of these findings and the potential implications for surgical decision making. Furthermore, the clinical follow up in this study may not have been reliable in highlighting the appearance of significant respiratory failure. The next step of the study will be to evidence demographic and clinical differences between pathological phrenic group versus normal phrenic group, in terms of age, gender, clinical phenotype, term of illness, disability, respiratory tests (spirometry, blood gas levels) by further statistical analysis.

In conclusion, the clinical relevance and prognostic value of diaphragm dysfunction measured by magnetic stimulation remain to be assessed; it must be kept in mind that surgeons should not be biased by the electrophysiological finding of an involvement of the cortico-diaphragmatic tract. Multicenter longitudinal studies, including larger numbers of CS and Syr patients, are needed to confirm our data.

## Figures and Tables

**Table 1:** Demographic and clinical features.

	Healthy Controls	Patient Groups			
Variables	*n* = 30	All *n* = 100	CS (Isolated) *n* = 34	CS + Syr *n* = 53	Syr (Isolated) *n* = 13
**Gender** (%)					
Male	11 (37)	25 (25)	5 (15)	14 (26)	6 (46)
Female	19 (63)	75 (75)	29 (85)	39 (74)	7 (54)
**Average age** (yrs ± SD)	48 ± 13.7	51 ± 13.08	49.7 ± 14.99	53 ± 11.91	55 ± 13.23
**Range of age** (yrs)	23–75	18–76	19–76	20–76	26–69
**Term of illness** (months)	-	110.96	106.56	111.59	107.91
**Clinical phenotype** (%)	-				
CS	-	34 (34)	34 (100)	-	-
Cervical Syr	-	22 (22)	-	16 (30)	6 (46)
Cervical-dorsal Syr	-	44 (44)	-	37 (70)	7 (54)
Syringobulbia (associated with cervical Syr)	-	8 (8)	-	7 (13)	1 (8)
**Clinical syndromes** (%)					
Myelopathy (sensory-motor-autonomic disorders)	-	61 (61)	7 (21)	41 (77)	13 (100)
Brainstem syndrome	-	43 (43)	22 (65)	21 (40)	0
Cerebellar syndrome	-	15 (15)	10 (29)	5 (9)	0

Percentages in brackets. CS = Chiari Syndrome; Syr = Syringomyelia/syringobulbia.

**Table 2:** Neurophysiological data: healthy controls and patients.

	Healthy Controls *n* = 30	All *n* = 100	CS *n* = 34	CS + Syr *n* = 53	Syr *n* = 13
CMCT right (ms)	5.9 ± 0.6	5.5 ± 0.8	5.7 ± 0.8	5.5 ± 0.8	n.e.
CMCT left (ms)	6.1 ± 0.7	5.6 ± 0.8	5.6 ± 0.7	5.5 ± 0.8	n.e.
CZ-MEP right latency (ms)	13.4 ± 0.8	14.2 ± 1.4	13.9 ± 1.3	14.3 ± 1.4	n.e.
CZ-MEP left latency (ms)	13.2 ± 1	14.16 ± 1.3	13.9 ± 1.3	14.2 ± 1.3	n.e.
C5-MEP right latency (ms)	7.3 ± 0.9	8.8 ± 1.2 **	8.3 ± 1 *	8.9 ± 1.2 **	9.5 ± 1.2 **
C5-MEP left latency (ms)	7.2 ± 1	8.7 ± 1.2 **	84 ± 1 *	8.8 ± 1.1 **	9.1 ± 1.5 **

Latency is reported in milliseconds (ms). CS = Chiari syndrome; Syr = syringomyelia; CMCT = central motor conduction time; CZMEP = cortical motor-evoked potential after magnetic stimulation; C5MEP = spinal motor-evoked potential; n.e. = not evaluable (CMCTs and CzMEP latencies were not calculated because Cz-MEPs were absent in Syr patients). Significant values (*p* < 0.05 * and *p* < 0.005 **) are highlighted in bold.

## Data Availability

All data are available on request to the corresponding author.
